# Effect of HIIT Training Modality in People with Pre-Diabetes

**DOI:** 10.3390/jfmk11010048

**Published:** 2026-01-22

**Authors:** Talia Tene, Raynier Zambrano-Villacres, Cristina Isabel Puruncajas-Rodríguez, Daniel Tettamanti Miranda, Mónica Cristina Tello-Moreno, Angela Priscila Campos-Moposita, Stalin Javier Caiza Lema, Martha Montalvan, Richard Tene-Fernandez

**Affiliations:** 1 Department of Chemistry, Universidad Técnica Particular de Loja, Loja 110160, Ecuador; 2 Dirección de Investigación, Universidad ECOTEC, Samborondón 092302, Ecuador; 3 Centro de Salud Tipo C Latacunga, Latacunga 070101, Ecuador; crisabelisabel@hotmail.es; 4 Carrera de Medicina, Universidad Católica de Santiago de Guayaquil, Guayaquil 090613, Ecuador; dantettamanti@gmail.com; 5 Pelvis Therapy Center, Ambato 180150, Ecuador; monicacristinatm@gmail.com; 6 Grupo de Investigación Nutrigenx, Universidad Técnica de Ambato, Ambato 180207, Ecuador; ap.campos@uta.edu.ec; 7 Carrera de Terapia Física, Facultad Ciencias de la Salud, Universidad Técnica de Ambato, Ambato 180207, Ecuador; sj.caiza@uta.edu.ec; 8 Escuela de Medicina, Universidad Espíritu Santo, Samborondón 0901952, Ecuador; mmontalvanmd53@gmail.com; 9 Hospital Metropolitano de Quito, Quito 170521, Ecuador; davidt-96@hotmail.com

**Keywords:** cardiorespiratory fitness, low-calorie diet, HIIT exercise, prediabetes

## Abstract

**Objectives**: Prediabetes is characterized by elevated blood glucose levels associated with insulin resistance, increasing the risk of progression to type 2 diabetes mellitus (T2DM) and cardiovascular disease. High-intensity interval training (HIIT) has emerged as an effective non-pharmacological strategy to improve insulin sensitivity and cardiorespiratory fitness. This study aimed to analyze the effects of HIIT alone or combined with a hypocaloric diet on metabolic and cardiorespiratory parameters in individuals with prediabetes. **Methods**: A controlled, longitudinal, single-blind intervention study enrolled 68 adults with prediabetes (mean age 42.22–46.60 years; 73.5% women) and randomized them to HIIT plus hypocaloric diet (n = 23), HIIT only (n = 23), or hypocaloric diet only (n = 22) for 13 weeks, with pre/post assessments of glucose, VO_2_max, blood pressure, FINDRISC. **Results**: Significant post-intervention differences were observed among groups in body mass index (*p* = 0.049), VO_2_max (*p* < 0.001), fasting glucose (*p* < 0.001), systolic blood pressure (*p* < 0.001), and diabetes risk (*p* = 0.038), with the greatest improvements consistently observed in Group A. In Group A, fasting glucose decreased from 111.94 to 91.28 mg/dL (−20.66 mg/dL; −18.5%), VO_2_max increased from 21.27 to 24.02 mL·kg^−1^·min^−1^ (+2.75; +12.9%), and systolic blood pressure decreased from 163.56 to 150.13 mmHg (−13.43 mmHg; −8.2%). No significant between-group differences were found for body weight (*p* = 0.271) or waist circumference (*p* = 0.174). **Conclusions**: HIIT combined with a hypocaloric diet is an effective and safe strategy for managing prediabetes, producing superior improvements in cardiorespiratory fitness, glycemic control, and reduction in diabetes risk compared with either intervention alone.

## 1. Introduction

Prediabetes is a condition marked by disproportionate increases in fasting plasma glucose (FPG), impaired glucose tolerance (IGT), and/or elevated glycated hemoglobin (HbA1c) levels [1]. The primary causes of IGT are insulin resistance (IR) and pancreatic β-cell dysfunction, which lead to higher postprandial glucose (PPG) levels. According to the American Diabetes Association (ADA), FPG levels of 100–125 mg/dL, 2-h PPG levels of 140–200 mg/dL, and HbA1c levels between 5.7% and 6.4% indicate prediabetes [2].

Prediabetes represents a major and growing public health challenge worldwide, affecting hundreds of millions of adults and substantially increasing the risk of progression to type 2 diabetes and cardiovascular disease. The high prevalence, frequent underdiagnosis, and substantial healthcare costs associated with dysglycemia underscore the need for scalable, preventive interventions [3,4].

Global data from the International Diabetes Federation (IDF) reported a 10.6% prevalence of adults with impaired glucose tolerance (IGT) in 2021, predicting an increase to 11.4% by 2045 [5]. In the United States, approximately 34.5% of adults are affected by prediabetes, according to the Centers for Disease Control and Prevention (CDC) [6]. Locally, data from the 2018 STEPS survey conducted by the World Health Organization (WHO) and the Ministry of Public Health (MSP) indicated around 727,000 cases of diabetes in Ecuador [7]. Additionally, the 2024 National Diabetes Prevalence Survey reported that one in every 18 Ecuadorians has diabetes, estimating a prevalence of 5.53% [8]; however, national data on prediabetes are still unavailable.

Although pharmacological approaches may be considered for selected high-risk individuals, their population-level impact is limited by eligibility criteria, long-term adherence, potential adverse effects, and cost, and they do not replace the foundational role of lifestyle modification. Consequently, time-efficient, evidence-based lifestyle strategies remain central to prediabetes management and diabetes prevention [9,10].

Given that prediabetes is the precursor to diabetes, lifestyle interventions—especially dietary changes and physical activity—are the main focus of primary prevention strategies to reduce its prevalence [11,12,13]. Among non-drug therapies, high-intensity interval training (HIIT) has become popular as a method that involves alternating periods of low, moderate, and high-intensity exercise with rest breaks [14]. Recent research [15] shows that HIIT protocols can improve lipid profiles and boost oxygen consumption (VO_2_) in the short term, highlighting advantages over moderate-intensity continuous training (MICT) [16,17,18,19]. Conversely, dietary patterns that involve low-calorie regimens can lead to improvements in blood sugar, metabolic health, and body composition [20]. Importantly, these changes—primarily due to reductions in visceral fat—can also result in a loss of skeletal muscle mass by 2% to 10%, making it crucial to choose the right training method for special populations [21].

However, although HIIT and hypocaloric diets show benefits as independent strategies, the evidence base remains limited regarding whether their combined implementation yields additive or synergistic improvements in glycemic control and cardiorespiratory fitness among adults with prediabetes. Therefore, whether HIIT plus a hypocaloric diet provides incremental benefits beyond either intervention alone remains insufficiently characterized [22,23].

The current paradigm raises an important question: Does combining HIIT with a hypocaloric diet produce additive or synergistic effects that are better than those from each intervention alone? This question is especially relevant in medical settings where time, resources, and patient adherence are limited. Therefore, the goal of this study was to examine the effects of a HIIT-based program in a population diagnosed with prediabetes.

## 2. Materials and Methods

### 2.1. Study Design

This study used a controlled, longitudinal, single-blind, three-arm parallel-group intervention design conducted over 13 weeks. Participants were randomly allocated to one of the intervention arms, and outcomes were assessed at baseline and post-intervention by evaluators blinded to group assignment. Participant and interventionist blinding was not feasible given the behavioral nature of the interventions; therefore, assessor blinding was implemented to minimize detection/measurement bias (single-blind design). Ethical principles from the Declaration of Helsinki were followed, and approval was obtained from the Bioethics and Biosafety Committee of the Technical University of Ambato (approval number 149-CEISH-UTA-2024). All participants gave written informed consent before enrollment. The sample size calculation was a priori, based on previous studies evaluating the effects of HIIT on VO_2_max and fasting glucose in individuals with prediabetes, an effect size of f = 0.30 was assumed, with an alpha level of 0.05 and a statistical power of 80%. The calculation indicated a minimum required sample size of 60 participants. To account for potential dropouts, a total of 68 participants were recruited.

### 2.2. Participants

**Figure 1:**
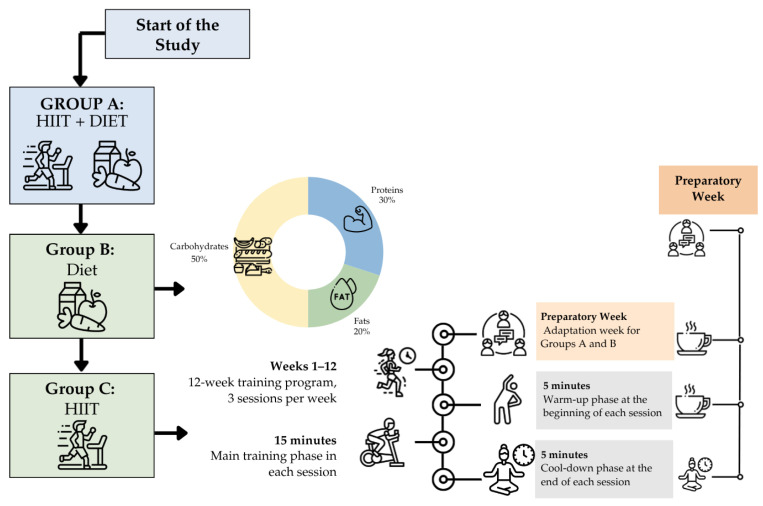
Research Protocol.

Sample size was determined a priori as described in Section 2.1. A total of 68 individuals attending the Type C Latacunga Health Center, part of District Health Unit 05D01 located in the canton of Latacunga, Cotopaxi Province, Zone 3, Ecuador, were included. A non-probabilistic convenience sampling method was used between July and October 2024, following approval from the Ethical Committee. Baseline physical-activity status was documented at enrollment (including whether participants engaged in organized sports or structured exercise) to reduce potential confounding from pre-intervention training status. Participants were randomly assigned into three study groups (Figure 1): Group A (HIIT plus hypocaloric diet, n = 23), Group B (HIIT only, n = 23), and Group C (hypocaloric diet only, n = 22). Randomization was performed using the online tool Random.org (List Randomizer: https://www.random.org/lists/, accessed on 7 December 2024), which generated an alphanumeric coded list to minimize selection bias across the groups. To ensure allocation concealment, the allocation list was generated and kept by a researcher not involved in recruitment or outcome assessment, and group assignment was revealed only after baseline measurements were completed. Due to the nature of the interventions, participant and exercise-supervisor blinding was not feasible; however, outcome assessors remained blinded throughout data collection. All participants underwent baseline and post-intervention assessments conducted by evaluators blinded to group assignment, maintaining a single-blind design.

### 2.3. Intervention

Generally, the HIIT sessions involved 4 to 8 high-intensity sets, lasting about 30 min in total, and included activities like running, cycling, and bodyweight exercises. Intensity levels ranged from submaximal to maximal capacity (80–95% of HRmax, 85% of peak power output [PPOmax], or 90% of VO_2_max), with recovery periods (50% of HRmax, 25% of PPOmax, or 30% of VO_2_max) [16]. Perceived exertion varied from “hard” to “very hard” (≥6 on the 10-point Borg scale or ≥15 on the 6–20 scale) [21]. Continuous monitoring during each session was advised [24].

Before the training program, an adaptation week was implemented for Groups A and B to provide instruction on the technical execution of exercises. A structured 12-week program was then developed, including moderate-volume HIIT (MV-HIIT) with 15 min of active work and moderate-interval HIIT (MI-HIIT) consisting of 30–120-s high-intensity bouts at 85% HR. Adherence to the prescribed exercise intensity was systematically monitored during each HIIT session using chest-strap heart rate monitors, ensuring that participants reached and maintained the target intensity zones (80–95% of HRmax during high-intensity intervals and 50% HRmax during recovery periods) estimated using the Karvonen equation [25,26]. Participants who failed to reach the target intensity in more than 20% of the intervals received individualized feedback and intensity adjustments in subsequent sessions. Overall session attendance and compliance with intensity prescriptions were recorded by physiotherapists.

The nutritional plan was divided into five meals and consisted of a hypocaloric diet of 1800 kcal [27]. Dietary adherence was assessed using weekly 24-h dietary recalls and structured food diaries completed by participants in Groups A and C. Adherence was considered acceptable when participants achieved ≥80% compliance with the prescribed caloric intake across the intervention period. The diet was normoproteic, normolipidic, and high in fiber, based on a moderate caloric deficit. A moderate reduction in carbohydrate intake from low-glycemic-index foods was recommended while maintaining adequate protein consumption (lean meats, legumes, low-fat dairy products, eggs), representing approximately 25–30% of total caloric intake. Fat intake ranged between 15–25%, fiber between 45–55%, and daily water consumption between 2.0 and 2.5 L, distributed throughout the day [28].

The Finnish Type 2 Diabetes Risk Score (FINDRISC) was applied to assess diabetes risk, collecting data on age, body mass index (BMI), waist circumference, physical activity, diet, history of antihypertensive medication use, previous hyperglycemia, and family history of type 2 diabetes mellitus (T2DM) [29]. Physical fitness was evaluated using the 6-Minute Walk Test (6MWT) and the Queen’s College Step Test, both classified as submaximal assessments of aerobic capacity (24).

The follow-up and supervision of the program were conducted during each session through structured interviews, carried out both in person and remotely on a weekly basis using digital platforms. These sessions were supervised by professionals specializing in physiotherapy, sports medicine, and nutrition.

### 2.4. Data Analysis

Data were processed using the SPSS statistical software package version 25.0, complemented by Microsoft Excel to establish a robust database that enabled detailed analysis.

Preliminary results of maximal oxygen consumption (VO_2_max) and submaximal heart rate (HRsubmax) were used to calculate training intensity, whereas final results revealed the effects on cardiorespiratory fitness, which were associated with fasting glucose indices.

Since both qualitative and quantitative data were collected, statistical tests were applied as follows: the Kolmogorov–Smirnov test to assess normality of distribution, Levene’s test to determine homogeneity of variances, one-way ANOVA for comparison among the three groups, and the Kruskal–Wallis test as a non-parametric method to verify the study hypothesis. In addition to *p*-values, effect sizes were calculated to quantify the magnitude of the intervention effects. For between-group comparisons performed using one-way ANOVA, partial eta squared (η^2^*p*) was reported as a measure of the overall effect of the intervention. For post hoc pairwise comparisons, Cohen’s d was calculated to estimate the magnitude of differences between specific groups. Effect sizes were interpreted as small (η^2^*p* = 0.01; d = 0.20), moderate (η^2^*p* = 0.06; d = 0.50), or large (η^2^*p* ≥ 0.14; d ≥ 0.80), according to established criteria.

## 3. Results

To begin, we analyzed and described the characteristics and distribution demographic variables, as shown in Table 1. Of the 68 participants randomly assigned to the three study groups, women predominated in Group A (15; 65.2%), Group B (22; 95.7%), and Group C (13; 59.1%) (*p* = 0.011). The mean ages were 46.60 (±7.649), 44.60 (±8.150), and 42.22 (±7.818) years for Groups A, B, and C, respectively (*p* = 0.139). Regarding educational level, most participants had completed high school education. Overall, no significant differences were observed in the distribution of educational levels among the groups (*p* = 0.728).

In terms of anthropometric characteristics, specifically in the between-group analysis of the measured variables, no significant differences were observed either before or after the intervention (*p* > 0.05), except for body mass index (BMI) after the intervention, which showed a *p*-value slightly below the alpha level (Table 2).

With respect to physiological parameters, the between-group analysis revealed significant differences after the intervention in submaximal heart rate (HRsubmax), systolic blood pressure (SBP), maximal oxygen consumption (VO_2_max), and fasting glucose (Table 3). In contrast, no significant differences were found between groups in the pre-intervention measurements (*p* > 0.05). Diastolic blood pressure (DBP) showed no statistically significant differences either before or after the intervention (*p* > 0.05).

In the between-group analysis of diabetes risk, no significant differences were observed before the intervention (*p* = 0.919), whereas significant differences emerged after the intervention (*p* = 0.038) (Table 4). The normality test showed significance levels below alpha (0.05) for fasting glucose (pre) (*p* = 0.029), weight (pre) (*p* = 0.000), weight (post) (*p* = 0.001), waist circumference (pre) (*p* = 0.033), and waist circumference (post) (*p* = 0.016), indicating that these variables did not meet the assumption of normality and therefore required non-parametric tests for statistical analysis between measurements. In contrast, VO_2_max (pre) (*p* = 0.200), VO_2_max (post) (*p* = 0.200), and fasting glucose (post) (*p* = 0.200) showed significance levels above 0.05, allowing the use of parametric tests for their analysis.

When comparing VO_2_max using ANOVA, a *p*-value of 0.000 was obtained, indicating a highly significant difference among the study groups after the intervention. The multiple comparison analysis of VO_2_max post-intervention revealed a substantial difference between Groups A and B—those who performed HIIT combined with the hypocaloric diet (*p* = 0.000) and HIIT alone (*p* = 0.000)—when compared with Group C, which followed only the diet. However, no significant difference was found between Groups A and B (*p* = 0.699).

The Kruskal–Wallis test indicated significant differences among the groups after the intervention in fasting glucose levels (*p* = 0.000) (Table 3), body mass index (*p* = 0.049) (Table 2), and diabetes risk (*p* = 0.038) (Table 4). Conversely, for weight (*p* = 0.271) and waist circumference (*p* = 0.174), *p*-values greater than alpha (0.05) suggested no significant differences among groups after the intervention (Table 3).

**Table 3:** Continuous outcomes before and after the intervention across groups (mean ± SD; fasting glucose: median ± SD).

Variables	Group A, n = 23	Group B, n = 23	Group C, n = 22	*p*-Value
Pre-weight, mean (SD) kg	67.26 (±10.032)	64.44 (±11.632)	70.64 (±14.348)	0.130
Post-weight, mean (SD) kg	59.16 (±9.056)	62.42 (±11.312)	65.24 (±13.632)	0.271
Pre-waist circumference, mean (SD) cm	90.06 (±0.082)	83.97 (±11.875)	89.32 (±14.448)	0.177
Post-waist circumference, mean (SD) cm	84.67 (±10.891)	80.10 (±13.377)	86.31 (±13.962)	0.174
Submax HR pre, mean (SD) bpm	141.43 (±7.378)	141.47 (±4.561)	140.95 (±4.874)	0.945
Submax HR post, mean (SD) bpm	156.19 (±7.396)	153.39 (±4.933)	141.09 (±5.254)	0.000
SBP pre, mean (SD) mmHg	163.56 (±11.015)	162.17 (±11.535)	163.90 (±11.027)	0.859
SBP post, mean (SD) mmHg	150.13 (±10.750)	150.04 (±10.679)	163.72 (±10.937)	0.000
DBP pre, mean (SD) mmHg	78.82 (±9.920)	76.78 (±10.361)	78.44 (±9.992)	0.593
DBP post, mean (SD) mmHg	77.26 (±9.478)	74.86 (±9.743)	77.31 (±10.157)	0.630
VO_2_max pre, mean (SD) mL/kg/min	21.27 (±1.271)	21.30 (±1.513)	21.65 (±1.048)	0.050
VO_2_max post, mean (SD) mL/kg/min	24.02 (±1.328)	23.56 (±1.450)	21.76 (±1.075)	0.000
Fasting glucose pre, median (SD) mg/dL	111.94 (±7.164)	113.72 (±6.616)	112.28 (±7.081)	0.658
Fasting glucose post, median (SD) mg/dL	91.28 (±5.337)	103.98 (±7.098)	97.39 (±6.992)	0.000

**Table 4:** FINDRISC type 2 diabetes risk category distribution before and after the intervention across groups (n, %).

Risk Category	Group A, n = 23	Group B, n = 23	Group C, n = 22	*p*-Value
Type 2 diabetes risk pre, n (%)				0.919
Low	0 (0.0%)	1 (4.3%)	1 (4.5%)	
Slightly elevated	3 (13.1%)	2 (8.7%)	3 (13.7%)	
Moderate	6 (26.1%)	5 (21.8%)	3 (13.7%)	
High	9 (39.1%)	13 (56.5%)	13 (59.1%)	
Very high	5 (21.7%)	2 (8.7%)	2 (9.0%)	
Type 2 diabetes risk post, n (%)				0.038
Low	2 (8.7%)	1 (4.3%)	0 (0.0%)	
Slightly elevated	11 (47.9%)	6 (26.2%)	6 (27.2%)	
Moderate	5 (21.7%)	10 (43.4%)	4 (18.3%)	
High	5 (21.7%)	5 (21.8%)	11 (50.0%)	
Very high	0 (0.0%)	1 (4.3%)	1 (4.5%)	

## 4. Discussion

High-intensity interval training (HIIT) as a treatment strategy for individuals with prediabetes has proven to be an effective approach for reducing blood glucose levels. This type of training improves insulin sensitivity, promotes weight loss, and lowers the risk of developing type 2 diabetes mellitus (T2DM). Furthermore, it enhances cardiovascular capacity and strengthens overall musculature, thereby improving metabolic health through short and efficient exercise sessions [17,30,31,32,33].

The prediabetic participants included in the present study had a similar mean age across all three groups (44–46 years) and a higher proportion of women. In all groups, most participants had completed secondary education. After the intervention, reductions in body weight and waist circumference were observed across the three groups, with greater but non-significant decreases in Group A (−8.10 kg ± 0.976 SD, *p* = 0.271) and waist circumference (−5.39 cm ± 0.859 SD, *p* = 0.174). Improvements were also noted in BMI among participants in Group A, who received HIIT combined with a hypocaloric diet, showing a significant decrease (*p* = 0.049) compared with Groups B and C. Similarly, Khodadadi et al. [34] reported that HIIT-based interventions led to changes in body composition among individuals with type 2 diabetes.

These findings are consistent with Liu et al. [19], who observed a significant reduction in body weight of 1.22 kg (95% CI: −2.23 to −0.18, *p* = 0.02) in patients with type 2 diabetes performing HIIT compared with those undergoing moderate-intensity training. A decrease in BMI of 0.85 kg/m^2^ (95% CI: −0.78 to −0.02, *p* = 0.04) was also reported. Likewise, Peng et al. [33] found that high-intensity, low-volume training led to reductions in body mass (RR = −0.94; 95% CI: −1.37 to −0.51; *p* < 0.0001) and BMI (RR = −0.31; 95% CI: −0.47 to −0.16; *p* < 0.0001). Since excess adiposity, particularly abdominal fat, is closely linked to insulin resistance, reductions in weight, waist circumference, and BMI may enhance insulin sensitivity, facilitating improved glycemic control. Additionally, weight loss and visceral fat reduction lower the risk of cardiovascular diseases and related complications.

When analyzing the physiological and metabolic parameters, the study showed overall improvements across the three groups, with the greatest increases observed in Group A—14.17 bpm (±0.018 SD) in HRsubmax and 2.75 mL/kg/min (±0.057 SD) in VO_2_max. Significant differences were observed between Groups A (*p* = 0.000) and B (*p* = 0.000) compared with Group C (*p* = 0.699), which did not show significant variation relative to the other groups. Similarly to Peng et al. [33], HIIT improved maximal oxygen uptake compared with the control group (RR = 5.45; 95% CI: 1.38–9.52; *p* = 0.009). Likewise, Liu et al. [19] reported moderate differences in relative VO_2_max (3.37 mL/kg/min, 95% CI: −0.55 to −0.19, *p* < 0.0001) and absolute VO_2_max (0.37 L/min, 95% CI: 0.28–0.45, *p* < 0.00001). These improvements may reduce cardiovascular risk and enhance quality of life, as higher VO_2_max values are associated with better insulin sensitivity and glucose regulation.

The magnitude of the VO_2_max increase in our HIIT plus hypocaloric diet group (+2.75 mL·kg^−1^·min^−1^) is consistent with meta-analytic estimates for HIIT in dysglycemic populations and comparable to reports under similar intensity and volume protocols [18,19]. Likewise, the reduction in fasting glucose (−20.66 mg/dL) falls within the range observed in interventions that combine high-intensity exercise with caloric restriction and in lifestyle programs targeting prediabetes [17,35]. Differences across studies are plausibly driven by protocol “dose” (interval intensity/duration and weekly volume), baseline cardiometabolic status, adherence, and supervision level [18,19,33,35].

An additional methodological strength is the single-blind, assessor-blinded design, which reduces detection and measurement bias in exercise–nutrition interventions where participant blinding is inherently infeasible and thereby supports the internal validity of the between-group differences.

HIIT likely elicits rapid improvements through intermittent high-intensity bouts that generate large glycogen flux and shear stress, activating AMPK–PGC-1α signaling, mitochondrial biogenesis, and GLUT4 translocation, alongside early endothelial and autonomic adaptations that support gains in VO_2_max and insulin sensitivity [18,19,33]. By contrast, MICT provides a sustained oxidative stimulus with greater total training time, promoting gradual increases in fat oxidation and aerobic enzyme activity, central adaptations via cumulative volume, and improvements in glycemic control primarily through increased energy expenditure [19,33]. In time-constrained adults with prediabetes, these distinct profiles help explain why HIIT can achieve comparable or greater cardiometabolic benefits with lower weekly volume, while MICT remains effective when higher volumes are feasible [17,19,33].

Systolic and diastolic blood pressure in Group A decreased by 15.43/1.56 mmHg (±0.3265/0.442), consistent with Peng et al. [33], who reported similar findings (RR = −4.01; 95% CI: −4.82 to −3.21; *p* < 0.0001). Fasting glucose levels also showed a greater post-intervention decrease in Group A compared with the other groups, with a reduction of 20.66 mg/dL (±1.827, *p* = 0.000). A significant improvement was also observed in diabetes risk (*p* = 0.038), which shifted from “very high” and “high” to “slightly elevated.” These main findings demonstrate that HIIT combined with a hypocaloric diet improves short-term glycemic control, consistent with the results of de Oliveira Teles et al. [17] and Arrieta-Leandro et al. [35]. In their studies, both authors reported reductions in glucose levels, supporting HIIT as a safe and effective option for managing glycemia and related metabolic factors.

Thus, the stimulus produced by the peaks of effort during HIIT sessions exerts a positive effect on energy metabolism by enhancing muscular glucose uptake. Moreover, scientific evidence indicates that HIIT not only improves short-term glycemic control but also produces lasting effects, suggesting that its benefits persist with continued adherence to the intervention.

### 4.1. Practical Implications

The training plan was designed to be simple and practical to improve exercise adherence and help participants build a consistent habit. With proper guidance and instruction from healthcare professionals, this routine can be easily repeated at home, as long as participants are aware of warning signs to avoid potential problems.

### 4.2. Limitations

Assessing respiratory capacity is crucial before starting any training program; therefore, primary healthcare—especially physiotherapy services—must include tools that enable such assessments. This ensures the reliability and validity of results, particularly when working with quantifiable population. While baseline physical-activity status was documented, prior structured exercise or organized sports participation was not strictly controlled, and residual confounding from pre-intervention training status cannot be fully excluded.

The 13-week duration of this study limits inferences about the durability and generalizability of the effects. Short-term gains in fasting glucose, VO_2_max, blood pressure, and risk scores may attenuate, plateau, or relapse after the supervised period, and maintenance strategies or adherence beyond week 13 were not assessed. Future studies should include 6–12-month follow-up to evaluate sustainability (including seasonal variation), weight-regain dynamics, glycemic variability, and cost-efficient support (e.g., remote monitoring) to preserve cardiometabolic benefits across diverse settings. Although participant blinding was not feasible, assessor blinding mitigated measurement bias and strengthened internal validity.

The use of non-probabilistic convenience sampling introduces potential selection bias that may affect external validity. Because recruitment occurred at a single center and relied on volunteers meeting our inclusion criteria, the sample may overrepresent motivated individuals, women, and participants with fewer comorbidities or greater readiness to change, while underrepresenting older adults, men, and less active or socioeconomically diverse groups. Consequently, the magnitude and consistency of the observed improvements may not fully generalize to broader prediabetes populations or other care settings. Future studies should consider multicenter, probability-based recruitment with stratification (e.g., by sex, age, and baseline activity) and reporting of responder/non-responder characteristics to enhance generalizability.

## 5. Conclusions

All three study groups—A (hypocaloric diet and HIIT), B (HIIT only), and C (hypocaloric diet only)—showed reductions in the anthropometric measures evaluated in this study, with Group A exhibiting the most significant decreases in body weight and waist circumference. Additionally, changes were observed in physiological and metabolic parameters, including reductions in blood pressure (BP) and increases in submaximal heart rate (HRsubmax) and maximum oxygen uptake (VO_2_max), especially in Group A, leading to improvements in cardiovascular health. Specifically, Group A demonstrated the greatest decrease in fasting glucose levels and a notable reduction in diabetes risk. The data show that combining exercise and diet is more effective than either intervention alone.

For routine prediabetes care, our findings support integrating supervised HIIT as 2–3 sessions per week for 12 weeks, using 1–4 min work intervals at ~80–95% HR_max interspersed with 1–2 min recovery at ~50–60% HR_max, with intensity monitored via heart-rate devices and session RPE. Concurrently, structured dietary counseling should target a moderate hypocaloric plan (~15–20% energy deficit) with brief weekly follow-ups (dietary recalls or food diaries). Primary care workflows can incorporate opportunistic screening (e.g., FINDRISC), referral to HIIT+diet programs with initial safety checks, and brief behavioral support (goal setting, reminders). To enhance scalability, programs may leverage group sessions, community facilities, and telemonitoring with low-cost wearables. Suggested implementation indicators include ≥80% session adherence and improvements in fasting glucose, VO_2_max, and diabetes risk scores at 12 weeks; health services and policymakers can pilot these bundles in primary care and evaluate costs and equity impacts for broader rollout.

## Figures and Tables

**Table 1:** Characteristics and distribution of demographic variables among the study groups.

Variables	Group A, n = 23	Group B, n = 23	Group C, n = 22	*p*-Value
**Sex, n (%)**				0.011
Women	15 (65.2%)	22 (95.7%)	13 (59.1%)	
Men	8 (34.8%)	1 (4.3%)	9 (40.9%)	
Age (SD ±)	46.60 (±7.649)	44.60 (±8.150)	42.22 (±7.818)	0.139
**Educational level, n (%)**				
Primary	0 (0.0%)	0 (0.0%)	0 (0.0%)	0.728
High school	12 (52.2%)	13 (56.5%)	10 (45.5%)	
Undergraduate	10 (43.5%)	7 (30.4%)	9 (40.9%)	
Graduate	1 (4.3%)	3 (13.1%)	3 (13.6%)	

SD ±: Standard deviation; *p*-value: Level of significance.

**Table 2:** BMI category distribution before and after the intervention across groups (n, %).

BMI Category	Group A, n = 23	Group B, n = 23	Group C, n = 22	*p*-Value
BMI pre, n (%)				0.887
Normal	5 (21.7%)	4 (17.4%)	4 (18.2%)	
Overweight	11 (47.8%)	13 (56.6%)	10 (45.4%)	
Obesity I	7 (30.5%)	5 (21.7%)	8 (36.4%)	
Obesity II	0 (0.0%)	1 (4.3%)	0 (0.0%)	
BMI post, n (%)				0.049
Normal	14 (60.8%)	8 (34.8%)	7 (31.8%)	
Overweight	9 (39.2%)	12 (52.2%)	12 (54.6%)	
Obesity I	0 (0.0%)	3 (13.0%)	3 (13.6%)	

## Data Availability

The original contributions presented in this study are included in the article. Further inquiries can be directed to the corresponding authors.
